# Hungarian Linguistic, Cross-Cultural and Age Adaptation of Transition Specific Questionnaires in Patients with Inflammatory Bowel Disease

**DOI:** 10.3390/children10040711

**Published:** 2023-04-12

**Authors:** Dóra Dohos, Alex Váradi, Nelli Farkas, Adrienn Erős, Katalin Eszter Müller, Anna Karoliny, Eszter Gombos, Éva Nemes, Noémi Vass, András Tárnok, Péter Hegyi, Patrícia Sarlós

**Affiliations:** 1 Institute for Translational Medicine, Medical School, University of Pécs, 12 Szigeti Street, HU-7624 Pécs, Hungary; 2 Szentágothai Research Centre, University of Pécs, 20 Ifjúság Street, HU-7624 Pécs, Hungary; 3 Heim Pál National Institute of Pediatrics, 86 Üllői Street, HU-1089 Budapest, Hungary; 4 Department of Family Care Methodology, Faculty of Health Science, Semmelweis University, 17 Vas Street, HU-1088 Budapest, Hungary; 5 Department of Pediatrics, Clinical Center, University of Debrecen, 98 Nagyerdei Boulevard, HU-4032 Debrecen, Hungary; 6 Albert Szent-Györgyi Clinical Center of Pediatrics and Child Health Centre, University of Szeged, 14-15, Korányi Street, HU-6725 Szeged, Hungary; 7 Department of Pediatrics, Medical School, University of Pécs, 7 József Attila Street, HU-7623 Pécs, Hungary; 8 Centre for Translational Medicine, Semmelweis University, 26 Üllői Street, HU-1085 Budapest, Hungary; 9 Division of Pancreatic Diseases, Heart and Vascular Center, Semmelweis University, 9 Gaál József Street, HU-1122 Budapest, Hungary; 10 Division of Gastroenterology, First Department of Medicine, Medical School, University of Pécs, 13 Ifjúság Street, 7624 Pécs, Hungary

**Keywords:** IBD, adaptation, transition, TRAQ, IBD–SES, STARx

## Abstract

**Objective:** In the TRANS–IBD clinical trial, the outcomes are measured with selected validated questionnaires. Cross-cultural and age adaptations of the Self-Efficacy Scale for adolescents and young adults (IBD–SES), the Transition Readiness Assessment Questionnaire (TRAQ), and the Self-Management and Transition Readiness Questionnaire (STARx) were performed. **Methods:** Linguistic and cultural adaptation was carried out with the usage of reliability coefficients (Cronbach’s α coefficients, Spearman’s rank correlation), and with confirmatory factor analysis (CFA; root Mean Square Error of Approximation [RMSEA], Comparative Fit Index [CFI], and Tucker-Lewis Index [TLI]). **Results:** 112 adolescents participated in the study (45.5% male, mean age 17 ± 1.98 years). CFA was acceptable in the IBD–SES and the TRAQ. Internal consistency was acceptable in IBD–SES and good in TRAQ (0.729; 0.865, respectively). Test–retest reliability was good in IBD–SES, but below the acceptable threshold in TRAQ (ρ = 0.819; ρ = 0.034). In STARx tools, RMSEA showed poor fit values, CFI and TLI were below acceptable fit values, and internal consistency was not satisfied (0.415; 0.693, respectively), while test–retest reliabilities were acceptable (ρ = 0.787; ρ = 0.788, respectively). **Conclusions:** Cross-cultural, age-specific adaptation was successfully completed with IBD–SES and TRAQ. Those are comparable to the original validated versions. The adaption of the STARx tools was not successful.

## 1. Introduction

Inflammatory bowel diseases (IBDs) are commonly diagnosed in adolescence and young adulthood [1,2,3,4]. In addition to the main therapeutic goals, such as symptom relief, remission, and prevention of complications, pediatricians should minimize the impact of illness and medications on growth and strive to normalize quality of life and psychosocial functioning [1,5].

Parents are deeply involved in the management of IBD in childhood, supporting and facilitating adherence to medication and scheduling appointments [6,7]. The central role and responsibility of parents in caring for their child’s illness are difficult to tolerate in their workplace, placing a financial and psychosocial burden on the family, which can generate a vicious circle for the disease to worsen [1,8,9]. Due to the nature of pediatric care, patients with IBD have shortcomings in their responsibilities for treating the disease [10]. Early education and the amelioration of self-management skills, provided by a multi-disciplinary team, are key to the success of the transition to adult health care [7].

In the case of a well-designed, planned, and dynamic transition, which has already received more positive feedback than a single-act transfer, is influenced by objective (e.g., multi-disciplinary, trained team, protocol-based management, financial difficulties) and subjective factors such as a patient’s self-management and knowledge of the disease [11,12,13,14]. The process should be adapted to the needs and abilities of the patients and their parents. This way, positive health care outcomes, appropriate self-care, and clinical and economic benefits could be guaranteed [15,16]. However, although the transition is a critical stage in the management of adolescents with chronic illnesses, the appropriate method, timing, and success rating factors are still unclear [17].

The transition process has been analysed on patients with different chronic conditions, but high-quality data on IBD are still missing [7,18], however, consensus statements have recently been developed for transition and better partnership between pediatric and adult gastroenterologists [19]. Therefore, we planned the ‘TRANS–IBD’ clinical trial [20] to evaluate the superiority of the most recommended method of transition, the joint visits [21,22]. After reviewing the Delphi studies, the outcomes of health care services, social life, and self-management have been analysed in our clinical trial [20,23,24,25]. Although self-report questionnaires have many limitations, to date, this is the easiest and cheapest way to evaluate patients’ opinions and subjective status. In addition to two originally non-transition-specific surveys, four transition-specific questionnaires are planned to use [20,26]. Self-efficacy has been measured using the Inflammatory bowel disease Self-Efficacy Scale for adolescents and young adults (IBD–SES), and transition readiness with the Transition Readiness Assessment Questionnaire (TRAQ), and the Self-Management and Transition Readiness Questionnaire (STARx) available for adolescents (STARx-A) and their parents (STARx-P) [17,27,28]. To date, there are no Hungarian adaptations of our targeted questionnaires.

Due to differences between nations, cross-cultural adaptation and ‘linguistically’ translated questionnaires are needed to maintain the original conceptual and content validity of the surveys to ensure a high standard of clinical trials and to provide comparable results to other foreign trials [29].

Therefore, we performed cross-cultural and age adaptation of the IBD–SES, TRAQ, and STARx questionnaires, as well as disease-specific adaptation for the TRAQ and STARx surveys. Young people with IBD aged 15–19 years were involved to achieve tools for the TRANS–IBD study and for future clinical practice.

## 2. Material and Methods

### 2.1. Population and Study Design

The adaptation was completed with 15–19-year-old IBD patients in nine hospitals in Hungary. All patients with IBD in the selected age group were involved. The data collection was performed between March 2020 and April 2021, the questionnaires were uploaded to our electronic database centrally. Baseline information was also gathered on personal and disease-related data such as gender, age, occupation, ethnicity, type of disease, activity, localization, previous medical history, surgery, and comorbidities.

### 2.2. Descriptions of the IBD–SES, TRAQ, and STARx Questionnaires

The IBD–SES questionnaire is a disease- and transition-specific tool to assess the self-efficacy of young adults diagnosed with IBD. It was validated by Izaguirre et al., and contains 13 questions (Q) with five answering options, scoring from one to five. Questions are structured in four domains, as follows: ‘managing medical care’ (Q1, 2, 3, 6, 8, 11); ‘managing everyday life with IBD’ (Q4, 9, 10); ‘managing feelings’ (Q7, 12); and ‘managing the future with IBD’ (Q5, 13) [27]. For Q3 and 5, unlike the others, the scores vary from five to one, thus ‘Completely agree’ means one. Due to the reverse scoring on Q3 and 5, the score ranges from 21 to 57, however, 13–65 points can be obtained by indicating the most negative and positive answers. Higher scores indicate higher self-efficacy. The authorization of our investigation was approved by Izaguirre via e-mail in January 2020.

TRAQ is a transition but non-disease-specific questionnaire that evaluates young people’s health and health care self-management skills. Originally Wood et al., validated the tool, which consisted of 20 questions on 5 structured topics, as follows: ‘managing medications’ (Q1–4); ‘appointment keeping’ (Q5–11); ‘tracking health issues’ (Q12–15); ‘talking with providers’ (Q16,17); ‘managing daily activities’ (Q18–20). Participants should choose the best fitting answer from the five possible ones to gain 1–5 points. The total score is derived from the averages of the points, so it ranges from one to five, with higher scores meaning higher transition readiness and self-management [28]. However, the demand for the survey adaptation and use permission has been re-sent since December 2019, but we did not receive any response until the adaptation process was completed.

STARx is a transition but not IBD-specific questionnaire measuring self-management and transition readiness of adolescents (STARx-A). This validated tool contains a version that assesses parents’ opinions about their children’s self-management and adulthood abilities (STARx-P). There is no difference between the content of the tools, only the personal pronunciation differs. In the original version, 18 questions are grouped into three categories to which patients should answer with one of the six options. The answers vary somewhat in the three categories. The questions could also be categorized into six domains according to the topic or knowledge measured, as follows: ‘medication management’ (Q2, 5, 8, 16); ‘provider communication’ (Q13, 14, 15); ‘engagement during appointments’ (Q1, 3, 4); ‘disease knowledge’ (Q10, 11, 12); ‘adult health responsibilities’ (Q17, 18); ‘resource utilization’ (Q6, 7, 9). The score ranges from 18 to 86, with a detailed scoring methodology provided by the author after approval [17]. Ferris endorsed our request to adapt and then use both STARx-A and STARx-P via e-mail in December 2019. 

### 2.3. Adaptation

The guideline of Beaton et al. was followed to perform cross-cultural and linguistic adaptation with two-way-translation and repeated testing methods [29]. The detailed methodology is demonstrated in Supplementary Figure S1.

### 2.4. Statistical Analyses

Descriptive statistics were used for demographic data. In the event of incomplete filling, the median values of the replies were used to fill the blank answers to the surveys, so that the questionnaire could be used during the complete analysis of the questionnaire.

In the case where data are non-normal due to the ordinal nature of the scale (e.g., a Likert-type scale is less than seven points) a proven good alternative, the weighted least squared method (WLSMV) estimator, was used [30]. The model fit to the original was evaluated with confirmatory factor analysis (CFA) including root mean square error of approximation (RMSEA), the comparative fit index (CFI) with a ≥0.90 cut-off limit, the Tucker–Lewis index (TLI) with a ≥0.90 cut off limit and the scaled Chi-Square. The RMSEA results below 0.08 were acceptable [31]. If more than 15% of patients signed extreme answers in the surveys, floor and/or ceiling effects could be identified.

Reliability indicates the consistency of the questionnaire in measuring the target topic, while internal consistency with Cronbach’s α coefficients measures the correlation between the items of the questionnaire. The minimum acceptable value was evaluated as α = 0.70, and the excellent value of internal consistency was determined if α ≥ 0.9. Furthermore, another parameter, the test–retest reliability with Spearman’s rank correlation evaluates the consistency of the results after the test was repeated on the same sample at different timepoints. The overall points of the surveys are used, and in this parameter ≥0.7 is statistically acceptable. The reliability is also determined by the expected stability of the construct to be analysed.

Correlations of demographic data and the survey’s total- and subscores were evaluated with Spearman’s rank correlation for continuous variables and Mann-Whitney U-test or Kruskal-Wallis rank sum test for categorical ones. The variables gender, age, disease duration, ethnicity, disease type, and treatments were estimated as potential influencing factors.

The threshold of significant results was *p* < 0.05. For all statistical analysis the R programming language (R Core Team, 2022, Vienna, Austria, R version 4.2) and the lavaan v0.6-12 R package were used [32,33].

## 3. Results

### 3.1. Characteristics of Patients Involved

A total of 114 adolescents with IBD were enrolled from four universities and five general hospitals, representing the whole of Hungary. The median number of participants involved was 10 (range 4–34). Two of the 114 adolescents were excluded due to incomplete questionnaires and the inability to self-report due to autism. Of the 112 patients analysed, 51 were male, and 61 were female, while 71 adolescents had CD, and 41 UC. The mean age was 17.00 ± 1.98, and the average duration of IBD was 3.61 ± 2.90 years. A total of 91.96% of the adolescents attended secondary school (Table 1).

### 3.2. The IBD-Self Efficacy Scale Questionnaire

#### 3.2.1. Questionnaire Description

During the adaptation, a minimal modification was performed (Supplementary Table S1). This was the shortest of the three questionnaires analysed, no unfilled surveys were received (Table 2A).

The floor effect was only observed in Q3 asking for regular medication intake, where, according to the parallel scoring system, point 5 represents the lowest score. The ceiling effect was observed for all items, but the highest was in Q7 and 13, where 51.7% and 87% of the participants who chose the answer ‘Completely agree’ achieved the maximum score, respectively. These two questions concerned subjective opinion and feelings (Q7: ‘When I am feeling frustrated about having IBD, I have someone I can turn to.’ Q13: ’I am hopeful that my IBD symptoms will get better.’). Q13 had to be excluded from the adaptation due to extremely oblique distribution and error in further analyses.

#### 3.2.2. Questionnaire Adequacy and Reliability

The theoretically based factor structure was verified by confirmatory factor analysis, but this was not completely feasible due to Q5 and 13, so these were excluded from the analyses. RMSEA yielded poor fit values, while CFI and TLI were statistically acceptable: (RMSEA: 0.101 [0.071–0.130]; CFI: 0.961; and TLI: 0.948) (Table 3).

The result of the internal consistency was acceptable (Cronbach’s α: 0.729) (Table 3). The analysis was also carried out separately in the domains. Due to the exclusion of Q5 and 13, three of the original four domains could be analysed. The values of the domains ranged from questionable to unacceptable (Cronbach’s α: 0.472–0.617) (Supplementary Table S2). The return rate of the re-test instrument was 63.4%, and test–retest reliability was rated as good (ρ = 0.819, *p* < 0.001) (Table 3).

#### 3.2.3. The Effect of Patient Demographics and Therapy on Self-Efficacy

A significantly higher total score was observed in patients of Hungarian ethnicity compared to other ethnicities, and in the group of those not receiving steroid treatment compared to patients on steroids (51 ± 8 vs. 46 ± 4, *p* = 0.016, and 51 ± 9 vs. 49 ± 5, *p* = 0.008, respectively). Scores in the ‘managing medical care’ domain were significantly higher in patients of Hungarian ethnicity compared to other ethnicities (24 ± 4 vs. 21 ± 2, respectively; *p* = 0.008). Subscores for the domain ‘managing everyday life with IBD’ were significantly higher in the steroid-naïve group than in patients with steroid treatment, and operated patients compared to patients without surgical intervention (44 ± 7 vs. 42 ± 4, *p* = 0.003, and 45 ± 7 vs. 44 ± 6, *p* = 0.034, respectively) (Supplementary Table S3).

### 3.3. The Transition Readiness Assessment Questionnaire

#### 3.3.1. Questionnaire Description

During the cross-cultural adaptation, changes had to be made to the questions related to the local insurance system (’Do you apply for health insurance if you lose your current coverage?’). In Hungary, health insurance for children is covered by the Hungarian State, and the insurance is provided for as long as the student relationship exists, regardless of age. (Supplementary Table S4) The distribution of responses is shown in Table 2B.

While the floor effect was achieved in only five items, the ceiling effect was reached in 15 questions. The most marked floor effect was observed in Q14 and 16, questioning patient–physician relationship, 59.8 and 69.6% of the participants answered with ‘I do not know, how to do it’. A notable ceiling effect was observed for Q3 and 12, where 74.5 and 51% of the patients chose the option ‘Yes, I always do it myself when it’s needed’. Q3 and 12 were related to IBD medication and careful self-documentation.

#### 3.3.2. Questionnaire Adequacy and Reliability

The RMSEA demonstrated a reasonable fit value, and the CFI, and TLI were statistically acceptable (RMSEA: 0.084 [CI: 0.068–0.101]; and CFI: 0.977; TLI: 0.972) (Table 3).

The value of Cronbach’s α was good (0.865) (Table 3). The analysis of internal consistency was performed separately for the five domains, and the results ranged between poor and good (Cronbach’s α: 0.546–0.827), (Supplementary Table S5). A total of 63.4% of the adolescents returned the retest survey on time, and the test–retest reliability result was below the acceptable threshold (ρ = 0.034, *p* = 0.779).

#### 3.3.3. The Effect of Patient Demographics and Therapy on Transition Readiness

Significantly higher scores were achieved by girls than boys in the third and fifth domains, called ‘tracking health issues’ and ‘managing daily activities’ (2.58 ± 1.15 vs. 2.89 ± 0.88, *p* = 0.044; and 3.41 ± 1.13 vs. 3.97 ± 0.78, *p* = 0.004, respectively). Significant correlations were observed between age and total score in domains 1–3. The older the patient, the higher score was achieved (ρ = 0.374, *p* < 0.001; ρ = 0.325, *p* < 0.001 in the medication management domain; ρ = 0.320, *p* = 0.001 in the appointment-keeping domain; and ρ = 0.362, *p* < 0.001 in the health issues tracking domains, respectively). In domain 3 ‘tracking health issues’, patients not receiving biological therapy scored significantly higher than patients on biologics (2.91 ± 1.01 vs. 2.52 ± 0.80, *p* = 0.041) (Supplementary Table S3).

### 3.4. The Self-Management and Transition Readiness Questionnaire

#### 3.4.1. Questionnaire Description

The pre-test process was conducted with both adolescents (STARx-A) and their parents (STARx-P) and resulted in minimal additional changes to the questionnaires (Supplementary Table S6). Any kind of modification was always performed in parallel to the questionnaires to preserve the original uniformity. Q4 and 15 have been deleted as they cannot be properly interpreted in our cultural environment and daily habits. Only 8.9% of the parents missed the fill-in. The distribution of STARx-A and STARx-P responses are shown in Table 2C,D.

The floor effect was reached for Q4 and 6 in both populations, where 49–62% of participants chose the answer ‘Never’. These questions relate to scheduling appointments and taking medication. A ceiling effect was observed at Q1, 5, 8–11, 13, 14, and 17 in both groups, and Q7 also reached a threshold in STARx-P. The most noticeable ceiling effect was evaluated for adolescents in Q1 and 10, and for adults in Q1, 10, 11; in these cases, more than 60% of the respondents chose the ‘Always’ option. These three questions measure knowledge about the disease.

#### 3.4.2. Questionnaire Adequacy and Reliability

RMSEA was a poor fit, and CFI and TLI results were almost but not quite at the threshold of acceptable fit values in the STARx-A and STARx-P questionnaires: (RMSEA: 0.123 [CI: 0.104–0.143] and 0.154 [CI: 0.134–0.173]; CFI: 0.865 and 0.878; TLI: 0.818 and 0.836, respectively) (Table 3).

The overall internal consistency of the STARx-A was unacceptable, and questionable in the STARx-P (Cronbach’s α: 0.415 and 0.693, respectively) (Table 3). In the six domains, Cronbach’s α results ranged from unacceptable to acceptable in STARx-A and STARx-P (0.014–0.779 and 0.297–0.828, respectively) (Supplementary Table S7). A total of 56.25% of the adolescents and adults returned the test, and the test–retest reliability was acceptable in both questionnaires (STARx-A: ρ = 0.787, *p* < 0.001; and STARx-P: ρ = 0.788, *p* < 0.001) (Table 3).

#### 3.4.3. The Effects of Patient Demographics and Therapy on Transition Readiness

The patients of Hungarian ethnicity scored significantly lower in domain 1 (compromising drug management) than adolescents of other ethnicities (13.87 ± 2.60 vs. 14.83 ± 0.75, respectively; *p* = 0.035). Disease duration was inversely related to domain 3 which deals with ‘engagement during appointments’ (ρ = 0.213, *p* = 0.032). Other disease characteristics such as therapy, prior surgery, and comorbidities were also examined, but no correlation with transition readiness was found (Supplementary Table S3).

## 4. Discussion and Conclusions

In the case of chronic diseases, patient transition is an extremely sensitive moment of patient management. Although the whole process is time-consuming and requires adequate preparation, various questionnaires are available to measure the adolescents’ disease-specific knowledge, self-efficacy, and transition readiness.

The IBD–SES questionnaire was validated for adolescent patients with IBD in the US and has not yet been adapted but used in Denmark [27,34,35]. Carlsen et al., designed and evolved a personalized transition program in which the IBD–SES questionnaire was identified as a validated patient-reported outcome measurement tool [35]. 

During the Hungarian adaptation and translation process no modifications were made. The measurement of the floor/ceiling effect can be strongly biased by the subjective aspect of self-assessment and self-opinion. At this stage of the adaptation process, we had to delete an item due to the prominent ceiling effect caused by an extreme oblique distribution. Finally, the analysis was performed with 11 of the original 13 items. The RMSEA values showed a poor fit, and CFI and TLI values demonstrated statistically acceptable model fit outcomes. The overall reliability score was acceptable, but the internal consistency results for the remaining three domains were not met; so, the domains should not be used separately, only as a whole. The test–retest reliability result was good. Our internal consistency and test–retest reliability results were as perfect as the values of Izaguirre et al. [34].

The self-efficacy of adolescents with IBD is important for adolescents’ quality of life. The different ethnic groups in Hungary may have segregational differences in quality of life and lifestyle and this can also be seen in their attitude toward this chronic disease. We assume these alterations were represented in the higher scores of the overall and ‘managing medical care’ domains in the Hungarian ethnicity compared to other ethnicities. Self-efficacy is also important for emotional quality of life since it is clearly influenced by negative and positive emotions. Patients who do not need steroid therapy or who already achieved a symptom-free life after surgery could cope with a more stressful situation and had a higher overall self-efficacy score and higher points in the ‘managing everyday life with IBD’ domain. The mean total score was relatively high in our population, which probably represents well-educated patients and high self-efficacy in managing their IBD.

The TRAQ survey was validated for adolescents with cystic fibrosis in the United States [28]. The questionnaire was used internationally, e.g., USA, England, Canada, and Italy, [28,36,37,38,39,40,41,42] and adapted for other chronic conditions, but an adaptation of IBD is still missing [11,43,44,45,46]. Adapted versions are available in Spanish for patients diagnosed with various chronic diseases, in Thai, and Brazilian-Portuguese for rheumatologic disorders, for the American pediatric population with epilepsy, and in Turkish for adolescents with diabetes [11,43,44,45,46].

Due to the differences in the Hungarian insurance system, we had to make modifications to the questionnaire. A Self-reported questionnaire can be easily biased by direct and indirect influencing factors and can be exponential when asking adolescents about their personal interactions and opinion. These possibly biased results of the floor-ceiling effect were present since a pronounced floor effect was found in questions about the patient-doctor relationship, while the ceiling effect appeared in the medication and self-documentation items.

The RMSEA values showed a reasonable fit, while the CFI and TLI values had acceptable model-fit outcomes. The reliability scores of the questionnaire showed good internal consistency, however, the internal consistency of the separated domains did not gain the satisfactory minimum in all cases. Thus, domains 3, 4, and 5 should not be used separately for comparison, only as a whole. The internal consistency values of the overall, and domains 1, 2, and 5, showed almost the same Cronbach’s α coefficient as in the original [28], and the overall internal consistency results were almost identical to the results of the adapted versions [43,44,45,46]. Internal consistency values for test–retest reliability remained below the acceptable threshold, and our result was lower than in other studies [43,45]. Despite the TRAQ’s high ranking of quality and utility, Carlsen et al. have also revealed that the questionnaire may not be developmentally appropriate for adolescents under 20 years [35,47]. According to our theory, the discrepancies from foreign results may be due to the altered repetition time, or other influencing factors, such as an overprotective family that has an influence on adolescents’ maturity. 

Gender and quality of life influence problem-solving and disease management skills and this alteration was seen in the girls’ and non-biological therapy group’s higher scores in disease-related problem domains. Based on a review by Johnson et al., age is one of the modifiable factors that should be considered to improve transition readiness [48]. The older the participants were, the higher the total score and subscore for domains self-management were achieved. The mean total score, which highlights important shortcomings of education and transition readiness, was in line with the scores of other adaptations [43,44].

The STARx tool was validated by Ferris et al. for adolescents with various chronic conditions in the US and [17], although not yet adapted, is used in different countries, such as Nigeria [49,50]. Ayuk et al. measured the pre-transition readiness of Nigerian adolescents with various chronic illnesses and concluded that regardless of age, transitional readiness and willingness were suboptimal [49]. Eluri et al. gathered that patients with eosinophilic esophagitis and eosinophilic gastroenteritis were less aware of the transition and their transition readiness was below that of patients with different conditions [50]. In addition, further Danish and Spanish versions of the questionnaires are available without published adaptation [51]. 

During our adaptation process, we modified the questionnaires to create the one that best fits our culture. Changing the number of items is not an uncommon practice in the cross-cultural adaptation process. A pronounced floor effect was observed for the items related to scheduling appointments and medication administration. These results may have come from the peculiarities of the Hungarian health service. During the confirmatory factor analysis, the RMSEA showed poor fit values, and the CFI and TLI results remained below the threshold of acceptable fit values in both cases. Overall reliability scores showed unacceptable and questionable results for STARx-A and STARx-P. Internal consistency scores reached acceptable minimums in STARx-A domain 2 and, STARx-P domains 2 and 4. These domains can be used separately for comparison, regardless of the results of the remaining domains and the whole. The validation of STARx-A was carried out separately for each age group, and our Cronbach’s α coefficients were slightly lower than the values in the 15–17 age group but lower than the overall values of the validation [17]. Furthermore, test–retest reliability was acceptable for both STARx-A and STARx-P. The overall score of STARx-P was close to STARx-A, indicating that parents’ efficiency and knowledge develop alongside their children’s [17].

Several strengths of our study can be mentioned. This is the first Hungarian adaptation of the questionnaires measuring transition readiness and self-efficacy, which process was guided by a strict methodology. Patients with various disease durations from several regions of Hungary were included, so the conclusions can be generalized. The study also has limitations, e.g., patients were only recruited from the largest gastroenterology centers, however, the IBD care is centralized in Hungary. Self-reported data were collected without objective measurement of the responses. Due to the pandemic in 2020 and 2021, only outpatients were recruited. The number of participants was less than expected, and the administration was done centrally due to the reduction of human resources. During the adaptation of the cross-sectional questionnaire, additional patient data, such as the detailed description of comorbidities, treatment, and social status were not included and evaluated. The rate of incomplete participation was low, but in these cases, the unanswered items were filled with median values to avoid exclusion. Due to the special pandemic and health care situation, it was not possible to determine the date of the re-test separately for the three questionnaires to avoid a low participation level.

To conclude, we performed a cross-cultural, age- and disease-specific adaptation of two transition readiness questionnaires, as well as a cross-cultural, and age-specific adaptation of a self-efficacy survey. According to our results, the Hungarian IBD–SES and TRAQ questionnaires are appropriate, trustworthy, reproducible, and comparable to the validated versions. Adapted, self-administered questionnaires that assess knowledge and skills, can help pediatricians identify the need for further education to gain autonomy [18].

Unfortunately, the adaptation of the STARx tools was not successful, due to the unacceptable results of the model fit analyses and internal consistency. Thus, future evaluations with Hungarian STARx tools cannot be compared with international results.

## Figures and Tables

**Table 1:** The main characteristics of patients involved.

Characteristics	Adolescents’ Population (n = 112)
male/female	51/61
ethnicity: Hungarian/other	105/7
age (mean ± SD; years)	17.00 ± 1.98
disease duration time (mean ± SD; years)	3.61 ± 2.90
Chron’s disease/ulcerative colitis	71/41
previous intestinal surgery (%)	13.39
comorbidities (%)	21.42
therapy (%): biologicals	41.96
steroids	24.10
azathioprine	36.66
5-ASA	58.92

Comorbidities: arthralgia 5.36%, hypertension 0.89%, asthma bronchiale 2.68%, ankylosing spondylitis 0.89%, celiac disease 0.89%, diabetes mellitus 1.78%, epilepsy 1.78%, reflux disease 0.89%, juvenile idiopathic arthritis 0.89%, lactose intolerance 0.89%, scoliosis 0.89%, polycystic ovary syndrome 0.89%, psoriasis vulgaris 0.89%, steatosis hepatis 0.89%, not defined 0.89%.

**Table 2:** Distribution of responses in IBD–SES (**A**); TRAQ (**B**); STARx (STARx-A: **C**, STARx-P: **D**).

(A) Distribution of Responses in IBD–SES						
Questions	Completely Disagree		Disagree	I Don’t Agree or Disagree	Agree	Completely Agree
(Q1) I understand what inflammatory bowel disease is	2 (1.8%)		1 (0.9%)	10 (8.9%)	44 (39.3%)	55 (49.1%)
(Q2) If someone asked me, I could explain what a colonoscopy is for	2 (1.8%)		3 (2.7%)	27 (24.1%)	34 (30.4%)	46 (41.1%)
(Q3) Remembering to take my IBD medications is hard	10 (8.9%)		11 (9.8%)	15 (13.4%)	38 (33.9%)	37 (33.0%)
(Q4) I can get through my day, even if I have symptoms like abdominal pain or fatigue	2 (1.8%)		4 (3.6%)	33 (29.5%)	44 (39.3%)	29 (25.9%)
(Q5)						
(Q6) When asked, I can remember the names of my current IBD medications and what they are used for	2 (1.8%)		5 (4.5%)	21 (18.8%)	33 (29.5%)	51 (45.5%)
(Q7) When I am feeling frustrated about having IBD, I have someone I can turn to	2 (1.8%)		3 (2.7%)	18 (16.1%)	31 (27.7%)	58 (51.8%)
(Q8) I feel comfortable talking to my IBD doctor about my questions or concerns	2 (1.8%)		2 (1.8%)	19 (17.0%)	36 (32.1%)	53 (47.3%)
(Q9) No matter where I am, I can find foods that I can eat	2 (1.8%)		11 (9.8%)	25 (22.3%)	32 (28.6%)	42 (37.5%)
(Q10) I know what to do when I think a flare is starting	2 (1.8%)		3 (2.7%)	32 (28.6%)	38 (33.9%)	37 (33.0%)
(Q11) I know where to find a reliable answer if I don’t understand what my IBD doctor tells me	7 (6.3%)		9 (8.0%)	36 (32.1%)	36 (32.1%)	24 (21.4%)
(Q12) I know what will make me feel better even when I am sad, frustrated, scared, angry, or annoyed	6 (5.4%)		5 (4.5%)	30 (26.8%)	40 (35.7%)	31 (27.7%)
(Q13)						
**(B) Distribution of responses in TRAQ**						
**Questions**	**I Don’t Know How to Do it**		**I Don** **’** **t Know, but I’d Like to Learn It**	**No, but I’ll Learn**	**Yes, I Started to Learn**	**Yes, I Always Do it Myself When It’s Needed**
(Q1) Do you fill a prescription if you need to?	8 (7.2%)		11 (9.8%)	26 (23.2%)	42 (37.5%)	23 (20.5%)
(Q2) Do you know what to do if you are having a bad reaction to your medications?	4 (3.6%)		13 (11.6%)	24 (21.4%)	47 (42.0%)	21 (18.8%)
(Q3) Do you take medications correctly and on your own?	2 (1.8%)		2 (1.8%)	4 (3.6%)	20 (17.9%)	82 (73.2%)
(Q4) Do you reorder medications before they run out?	7 (6.3%)		11 (9.8%)	27 (24.1%)	33 (29.5%)	32 (28.6%)
(Q5) Do you call the doctor’s office to make an appointment?	15 (13.4%)		11 (9.8%)	54 (48.2%)	23 (20.5%)	6 (5.4%)
(Q6) Do you follow up on any referrals for tests or check-ups or labs?	5 (4.5%)		5 (4.5%)	13 (11.6%)	56 (50.0%)	31 (27.7%)
(Q7) Do you arrange for your ride to medical appointments?	8 (7.2%)		11 (9.8%)	31 (27.7%)	25 (22.3%)	35 (31.3%)
(Q8) Do you call the doctor about unusual changes in your health (e.g., allergic reactions)?	4 (3.6%)		6 (5.4%)	19 (17.0%)	30 (26.8%)	49 (43.8%)
(Q9) Do you apply for health insurance if you lose your current coverage?	11 (9.8%)		9 (8.0%)	27 (24.1%)	25 (22.3%)	36 (32.1%)
(Q10) Do you know what your health insurance covers?	19 (17.0%)		21 (18.8%)	27 (24.1%)	30 (26.8%)	13 (11.6%)
(Q11) Do you manage your money and budget household expenses (e.g., use a checking/debit card)?	15 (13.4%)		11 (9.8%)	24 (21.4%)	37 (33.0%)	23 (20.5%)
(Q12) Do you fill out the medical history form, including a list of your allergies?	5 (4.5%)		16 (14.3%)	19 (17.0%)	13 (11.6%)	58 (51.8%)
(Q13) Do you keep a calendar or list of medical and other appointments?	35 (31.3%)		5 (4.5%)	18 (16.1%)	16 (14.3%)	37 (33.0%)
(Q14) Do you make a list of questions before the doctor’s visit?	67 (59.8%)		5 (4.5%)	16 (14.3%)	16 (14.3%)	7 (6.3%)
(Q15) Do you get financial help with school or work?	78 (69.6%)		4 (3.6%)	2 (1.8%)	2 (1.8%)	25 (22.3%)
(Q16) Do you tell the doctor or nurse what you are feeling?	24 (21.4%)		35 (31.3%)	22 (19.8%)	20 (18%)	9 (8.0%)
(Q17) Do you answer questions that are asked by the doctor, nurse or clinic staff?	11 (9.8%)		15 (13.4%)	18 (16.1%)	38 (34.2%)	27 (24.1%)
(Q18) Do you help plan or prepare meals/food?	6 (5.4%)		14 (12.6%)	19 (17.0%)	46 (41.1%)	25 (22.3%)
(Q19) Do you keep your home/room clean or clean up after meals?	5 (4.5%)		8 (7.2%)	22 (19.6%)	29 (26.1%)	46 (41.1%)
(Q20) Do you use neighbourhood stores and services (e.g., grocery stores and pharmacy stores)?	5 (4.5%)		12 (10.7%)	20 (18%)	38 (34.2%)	35 (31.3%)
**(C) Distribution of responses in STARx-A**						
**Questions**	**Never**	**Almost Never**	**Sometimes**	**Almost Always**	**Always**	**I Do Not Take Any Medicine**
(Q1) How often did you make an effort to understand what your doctor told you?	3 (2.7%)	1 (0.9%)	4 (3.6%)	31 (27.7%)	71 (63.4%)	
(Q2) How often did you take your medicines on your own?	2 (1.8%)	1 (0.9%)	5 (4.5%)	31 (27.7%)	68 (60.7%)	5 (4.5%)
(Q3) How often did you ask doctors or nurses questions about your illness, medicines, or medical care?	11 (9.8%)	16 (14.3%)	55 (49.1%)	21 (18.8%)	8 (7.1%)	
(Q4)						
(Q5) How often did you need someone to remind you to take your medicines?	28 (25.0%)	27 (24.1%)	42 (37.5%)	7 (6.3%)	3 (2.7%)	4 (3.6%)
(Q6) How often did you use things like pillboxes, schedules, or alarms to help you take your medicines when they were supposed to?	55 (49.1%)	17 (15.2%)	16 (14.3%)	9 (8.0%)	9 (8.0%)	5 (4.5%)
(Q7) How often did you use the internet, books, or other guides to find out more about his/her illness?	14 (12.5%)	15 (13.4%)	56 (50.0%)	19 (17.0%)	8 (7.1%)	
(Q8) How often did you forget to take your medicines?	28 (25.0%)	39 (34.8%)	34 (30.4%)	4 (3.6%)	3 (2.7%)	4 (3.6%)
(Q9) How often did you work with your doctor to take care of new health problems that came up?	10 (8.9%)	7 (6.3%)	29 (25.9%)	31 (27.7%)	35 (31.3%)	
	**Nothing**	**Not much**	**A little**	**Some**	**A lot**	**I do not take any medicine**
(Q10) How much do you know about your illness?	0 (0.0%)	2 (1.8%)	7 (6.3%)	30 (26.8%)	73 (65.2%)	0 (0.0%)
(Q11) How much do you know about taking care of your illness?	0 (0.0%)	5 (4.5%)	9 (8.0%)	42 (37.5%)	56 (50.0%)	
(Q12) How much do you know about what will happen if you do not take your medicines?	5 (4.5%)	5 (4.5%)	11 (9.8%)	27 (24.1%)	59 (52.7%)	5 (4.5%)
	**Very hard**	**Somewhat hard**	**Neither hard nor easy**	**Somewhat Easy**	**Very easy**	**I do not take any medicine**
(Q13) How easy or hard is it for you to talk to your doctor?	0 (0.0%)	3 (2.7%)	11 (9.8%)	39 (34.8%)	59 (52.7%)	
(Q14) How easy or hard is it for you to make a plan with your doctor to care for your health?	1 (0.9%)	1 (0.9%)	13 (11.6%)	46 (41.1%)	51 (45.5%)	
(Q15)						
(Q16) How easy or hard is it for you to take your medicines the way they are supposed to?	1 (0.9%)	0 (0.0%)	10 (8.9%)	38 (33.9%)	56 (50.0%)	6 (5.4%)
(Q17) How easy or hard is it for you to take care of yourself?	3 (2.7%)	4 (3.6%)	22 (19.6%)	46 (41.1%)	37 (33.0%)	
(Q18) How easy or hard do you think it will be for you to move from pediatrics to adult-focused care?	11 (9.8%)	26 (23.2%)	39 (34.8%)	29 (25.9%)	7 (6.3%)	
**(D) Distribution of responses in STARx-P**						
**Questions**	**Never**	**Almost Never**	**Sometimes**	**Almost Always**	**Always**	**Not Needed for My Child’s Care**
(Q1) How often did your child make an effort to understand what his/her doctor told them?	2 (2.0%)	1 (1.0%)	10 (9.8%)	26 (25.5%)	63 (61.8%)	
(Q2) How often did your child take his/her medicines on their own?	2 (2.0%)	4 (4.0%)	7 (6.9%)	36 (35.3%)	49 (48.0%)	4 (3.6%)
(Q3) How often did your child ask his/her doctor or nurse questions about their illness, medicines, or medical care?	6 (5.9%)	9 (8.8%)	55 (54.0%)	22 (21.6%)	10 (9.8%)	
(Q4)						
(Q5) How often did your child need someone to remind him/her to take their medicines?	25 (24.5)	24 (23.5%)	39 (38.2%)	8 (7.8%)	2 (2.0%)	3 (3.0%)
(Q6) How often did your child use things like pillboxes, schedules, or alarm clocks to help him/her take their medicines when they were supposed to?	52 (51.0%)	7 (6.9%)	20 (19.6%)	9 (8.8%)	10 (9.8%)	4 (4.0%)
(Q7) How often did your child use the internet, books, or other guides to find out more about his/her illness?	13 (12.7%)	7 (6.9%)	49 (48.0%)	17 (16.6%)	16 (15.7%)	
(Q8) How often did your child forget to take his/her medicines?	29 (28.4%)	26 (25.5%)	40 (39.2%)	2 (2.0%)	1 (1.0%)	4 (4.0%)
(Q9) How often did your child work with his/her doctor to take care of new health problems that came up?	4 (4.0%)	7 (6.9%)	31 (30.4%)	22 (21.6%)	38 (37.3%)	
	**Nothing**	**Not much**	**A little**	**Some**	**A lot**	**Not needed for my child’s care**
(Q10) How much does your child know about his/her illness?	0 (0.0%)	1 (1.0%)	3 (3.0%)	17 (16.6%)	79 (77.5%)	
(Q11) How much does your child know about taking care of his/her illness?	0 (0.0%)	2 (2.0%)	5 (4.9%)	21 (20.6%)	72 (70.6%)	
(Q12) How much does your child know about what will happen if he/she does not take their medicines?	0 (0.0%)	2 (2.0%)	4 (4.0%)	29 (28.4%)	61 (59.8%)	4 (4.0%)
	**Very hard**	**Somewhat hard**	**Neither hard nor easy**	**Somewhat easy**	**Very easy**	**Not needed for my child’s care**
(Q13) How easy or hard is it for your child to talk to his/her doctor?	0 (0.0%)	2 (2.0%)	14 (13.7%)	36 (32.1%)	47 (46.1%)	
(Q14) How easy or hard is it for your child to make a plan with his/her doctor to care for his/her health?	1 (1.0%)	2 (2.0%)	16 (15.7%)	44 (43.1%)	35 (34.3%)	
(Q15)						
(Q16) How easy or hard is it for your child to take his/her medicines like they are supposed to?	2 (2.0%)	3 (3.0%)	8 (7.8%)	28 (27.5%)	54 (52.9%)	4 (4.0%)
(Q17) How easy or hard is it for your child to take care of himself/herself?	4 (4.0%)	5 (4.9%)	15 (14.7%)	41 (40.2%)	34 (33.3%)	
(Q18) How easy or hard do you think it will be for your child to move from pediatrics to adult-focused care?	11 (10.8%)	17 (16.6%)	31 (30.4%)	31 (30.4%)	9 (8.8%)	

**Table 3:** Indices of IBD–SES, TRAQ, STARx questionnaire adequacy and reliability.

INDICES	IBD–SES Questionnaire	TRAQ Questionnaire	STARx-A Questionnaire	STARx-P Questionnaire
number of responses (n)	112	111	112	102
total score, mean (SD)	44 (±6.4)	3.4 (±0.7)	60 (±5.5)	56 (±16.8)
CFI	0.961	0.977	0.865	0.878
TLI	0.948	0.972	0.818	0.836
RMSEA (CI)	0.101 (0.071–0.130)	0.084 (0.068–0.101)	0.123 (0.104–0.143)	0.154 (0.134–0.173)
*Cronbach’s α*	0.729	0.865	0.415	0.693
number or retests (n)	69	71	70	69
retest total score, mean (SD)	42 (±9.7)	3.3 (±0.7)	59 (±7.5)	58 (±8.9)
test-retest: ρ (*p*)	0.819 (<0.001)	0.034 (0.779)	0.787 (<0.001)	0.778 (<0.001)

n: number; SD: standard deviation; CFI: comparative fit index, TLI: Tucker-Lewis index, CI: confidence interval.

## Data Availability

The data underlying this article is available in the article and in its online Supplementary Material.

## Supplementary Materials

The following supporting information can be downloaded at: https://www.mdpi.com/article/10.3390/children10040711/s1, Figure S1: Steps of adaptation; Table S1: The Hungarian version of the IBD–SES questionnaire [52]; Table S2: The mean and the Cronbach’s α values of the domains in IBD–SES; Table S3: Correlation between total and domain subscores of the questionnaires and demographic data; Table S4: The Hungarian version of the TRAQ questionnaire [52]; Table S5: The mean and the Cronbach’s α values of the domains in TRAQ; Table S6: The Hungarian version of the STARx questionnaire (STARx-A: A; STARx-P: B) [52]; Table S7: The mean and the Cronbach’s α values of the domains in STARx.

## Abbreviations

CFI	Comparative fit index
IBD	Inflammatory bowel disease
IBD–SES	IBD Self-Efficacy Scale for adolescents and young adults
Q	question
RMSEA	Root mean square error of approximation
STARx-A	Self-Management and Transition Readiness Questionnaire (Adolescent Version)
STARx-P	Self-Management and Transition Readiness Questionnaire (Parent Version)
TLI	Tucker-Lewis index
TRAQ	Transition Readiness Assessment Questionnaire

## References

[1] Rosen M.J., Dhawan A., Saeed S.A. (2015). Inflammatory Bowel Disease in Children and Adolescents. JAMA Pediatr..

[2] Baldassano R.N., Piccoli D.A. (1999). Inflammatory bowel disease in pediatric and adolescent patients. Gastroenterol. Clin. N. Am..

[3] Sýkora J., Pomahačová R., Kreslová M., Cvalínová D., Štych P., Schwarz J. (2018). Current global trends in the incidence of pediatric-onset inflammatory bowel disease. World J. Gastroenterol..

[4] Müller K.E., Lakatos P.L., Arató A., Kovács B.J., Várkonyi Á., Szűcs D., Szakos E., Sólyom E., Kovács M., Polgár M. (2013). Incidence, Paris classification, and follow-up in a nationwide incident cohort of pediatric patients with inflammatory bowel disease. J. Pediatr. Gastroenterol. Nutr..

[5] Greenley R.N., Hommel K.A., Nebel J., Raboin T., Li S.H., Simpson P., Mackne L. (2010). A meta-analytic review of the psychosocial adjustment of youth with inflammatory bowel disease. J. Pediatr. Psychol..

[6] Cushman G., Shih S., Reed B. (2020). Parent and Family Functioning in Pediatric Inflammatory Bowel Disease. Children.

[7] (2014). Abraham BP, Kahn SA Transition of Care in Inflammatory Bowel Disease. Gastroenterol. Hepatol..

[8] Mehta F. (2016). Report: Economic implications of inflammatory bowel disease and its management. Am. J. Manag. Care.

[9] Taft T.H., Keefer L. (2016). A systematic review of disease-related stigmatization in patients living with inflammatory bowel disease. Clin. Exp. Gastroenterol..

[10] Gumidyala A.P., Greenley R.N., Plevinsky J.M., Poulopoulos N., Cabrera J., Lerner D., Noe J.D., Walkiewicz D., Werlin S., Kahn S.A. (2018). Moving On: Transition Readiness in Adolescents and Young Adults With IBD. Inflamm. Bowel Dis..

[11] De Cunto C.L., Eymann A., de Los Ángeles Britos M., González F., Roizen M., de Las Mercedes Rodríguez Celin M., Guppy E.S. (2017). Cross-cultural adaptation of the Transition Readiness Assessment Questionnaire to Argentinian Spanish. Arch. Argent Pediatr..

[12] Goodhand J., Dawson R., Hefferon M., Tshuma N., Swanson G., Wahed M., Croft N.M., Lindsay J.O. (2010). Inflammatory bowel disease in young people: The case for transitional clinics. Inflamm. Bowel Dis..

[13] Otto C., Tárnok A., Erős A., Szakács Z., Vincze Á., Farkas N., Sarlós P. (2019). Planned Transition of Adolescent Patients with Inflammatory Bowel Disease Results in Higher Remission Rates. J. Pediatr. Nurs..

[14] Callahan S.T., Winitzer R.F., Keenan P. (2001). Transition from pediatric to adult-oriented health care: A challenge for patients with chronic disease. Curr. Opin. Pediatr..

[15] Rohatinsky N., Risling T., Hellsten L.A.M., Kumaran M. (2020). Inflammatory Bowel Disease Nurses’ Perspectives: Prioritizing Adolescent Transition Readiness Factors. J. Pediatr. Nurs..

[16] Schütz L., Radke M., Menzel S., Däbritz J. (2019). Long-term implications of structured transition of adolescents with inflammatory bowel disease into adult health care: A retrospective study. BMC Gastroenterol..

[17] Ferris M., Cohen S., Haberman C., Javalkar K., Massengill S., Mahan J.D., Kim S., Bickford K., Cantu G., Medeiros M. (2015). Self-Management and Transition Readiness Assessment: Development, Reliability, and Factor Structure of the STARx Questionnaire. J. Pediatr. Nurs..

[18] Sawicki G.S., Lukens-Bull K., Yin X., Demars N., Huang I.-C., Livingood W., Reiss J., Wood D. (2011). Measuring the transition readiness of youth with special healthcare needs: Validation of the TRAQ--Transition Readiness Assessment Questionnaire. J. Pediatr. Psychol..

[19] Kumagai H., Shimizu T., Iwama I., Hagiwara S.-I., Kudo T., Takahashi M., Saito T., Kunisaki R., Uchino M., Hiraoka S. (2022). A consensus statement on health-care transition for childhood-onset inflammatory bowel disease patients. Pediatr. Int..

[20] Erős A., Dohos D., Veres G., Tárnok A., Vincze Á., Tészás A., Zádori N., Gede N., Hegyi P., Sarlós P. (2020). Effect of joint transition visits on quality of life in adolescents with inflammatory bowel diseases: A protocol for a prospective, randomised, multicentre, controlled trial (TRANS-IBD). BMJ Open.

[21] van Rheenen P.F., Aloi M., Biron I.A., Carlsen K., Cooney R., Cucchiara S., Cullen G., Escher J.C., Kierkus J., Lindsay J.O. (2017). European Crohn’s and Colitis Organisation Topical Review on Transitional Care in Inflammatory Bowel Disease. J. Crohns. Colitis..

[22] Brooks A.J., Smith P.J., Cohen R., Collins P., Douds A., Forbes V., Gaya D.R., Johnston B.T., McKiernan P.J., Murray C.D. (2017). UK guideline on transition of adolescent and young persons with chronic digestive diseases from paediatric to adult care. Gut.

[23] Suris J.C., Akre C. (2015). Key elements for, and indicators of, a successful transition: An international Delphi study. J. Adolesc. Health.

[24] Fair C., Cuttance J., Sharma N., Maslow G., Wiener L., Betz C., Porter J., McLaughlin S., Gilleland-Marchak J., Renwick A. (2016). International and Interdisciplinary Identification of Health Care Transition Outcomes. JAMA Pediatr..

[25] van den Brink G., van Gaalen M.A.C., de Ridder L., van der Woude C.J., Escher J.C. (2019). Health Care Transition Outcomes in Inflammatory Bowel Disease: A Multinational Delphi Study. J. Crohns. Colitis..

[26] Dohos D., Váradi A., Farkas N., Erős A., Párniczky A., Schäfer E., Kosaras É., Czelecz J., Hegyi P., Sarlós P. (2022). Hungarian Linguistic, Cross-Cultural, and Age Adaptation of the Patient Satisfaction with Health Care in Inflammatory Bowel Disease Questionnaire (CACHE) and the Medication Adherence Report Scale (MARS). Children.

[27] Izaguirre M.R., Keefer L. (2014). Development of a self-efficacy scale for adolescents and young adults with inflammatory bowel disease. J. Pediatr. Gastroenterol. Nutr..

[28] Wood D., Rocque B., Hopson B., Barnes K., Johnson K.R. (2019). Transition Readiness Assessment Questionnaire Spina Bifida (TRAQ-SB) specific module and its association with clinical outcomes among youth and young adults with spina bifida. J. Pediatr. Rehabil. Med..

[29] Beaton D.E., Bombardier C., Guillemin F., Ferraz M.B. (2000). Guidelines for the process of cross-cultural adaptation of self-report measures. Spine.

[30] Chen P.Y., Yang C.M., Morin C.M. (2015). Validating the cross-cultural factor structure and invariance property of the Insomnia Severity Index: Evidence based on ordinal EFA and CFA. Sleep Med..

[31] Bentler P.M. (1999). Cutoff criteria for fit indexes in covariance structure analysis: Conventional criteria versus new alternatives. Struct. Equ. Model.

[32] R Core Team (2021). A Language and Environment for Statistical Computing.

[33] Rosseel Y. (2012). Lavaan: An R Package for Structural Equation Modeling. J. Stat. Softw..

[34] Izaguirre M.R., Taft T., Keefer L. (2017). Validation of a Self-efficacy Scale for Adolescents and Young Adults With Inflammatory Bowel Disease. J. Pediatr. Gastroenterol. Nutr..

[35] Carlsen K., Hald M., Dubinsky M.C., Keefer L., Wewer V. (2019). A Personalized eHealth Transition Concept for Adolescents with Inflammatory Bowel Disease: Design of Intervention. JMIR Pediatr. Parent..

[36] Rague J.T., Kim S., Hirsch J.A., Meyer T., Rosoklija I., Larson J.E., Swaroop V.R., Bowman R.M., Bowen D.K., Cheng E.Y. (2021). Assessment of Health Literacy and Self-reported Readiness for Transition to Adult Care Among Adolescents and Young Adults with Spina Bifida. JAMA Netw. Open.

[37] Feingold J.H., Kaye-Kauderer H., Mendiolaza M., Dubinsky M.C., Keefer L., Gorbenkoc K. (2021). Empowered transitions: Understanding the experience of transitioning from pediatric to adult care among adolescents with inflammatory bowel disease and their parents using photovoice. J. Psychosom. Res..

[38] Arvanitis M., Hart L.C., DeWalt D.A., de Ferris M.E.D.-G., Sawicki G.S., Long M.D., Martin C.F., Kappelman M.D. (2021). Transition Readiness Not Associated With Measures of Health in Youth With IBD. Inflamm. Bowel Dis..

[39] Chan J.T., Soni J., Sahni D., Mantis S., Boucher-Berry C. (2019). Measuring the Transition Readiness of Adolescents With Type 1 Diabetes Using the Transition Readiness Assessment Questionnaire. Clin. Diabetes.

[40] Livanou M., Lane R. (2021). Assessing the Feasibility of a Multicenter Transition Intervention Model Across Adolescent Secure Services in England (MOVING FORWARD): Protocol for a Feasibility Cluster Randomized Controlled Trial. JMIR Res. Protoc..

[41] Cleverley K., Stevens K., Davies J., McCann E., Ashley T., Brathwaite D., Gebreyohannes M., Nasir S., O’Reilly K., Bennett K.J. (2021). Mixed-methods study protocol for an evaluation of the mental health transition navigator model in child and adolescent mental health services: The Navigator Evaluation Advancing Transitions (NEAT) study. BMJ Open.

[42] Corsello A., Pugliese D., Bracci F., Knafelz D., Papadatou B., Aloi M., Cucchiara S., Guidi L., Gasbarrini A., Armuzzi A. (2021). Transition of inflammatory bowel disease patients from pediatric to adult care: An observational study on a joint-visits approach. Ital. J. Pediatr..

[43] Kittivisuit S., Lerkvaleekul B., Soponkanaporn S., Ngamjanyaporn P., Vilaiyuk S. (2021). Assessment of transition readiness in adolescents in Thailand with rheumatic diseases: A cross-sectional study. Pediatr. Rheumatol. Online J..

[44] Clark S.J., Beimer N.J., Gebremariam A., Fletcher L.L., Patel A.D., Carbone L., Guyot J.A., Joshi S.M. (2020). Validation of EpiTRAQ, a transition readiness assessment tool for adolescents and young adults with epilepsy. Epilepsia Open.

[45] Kızıler E., Yıldız D., Fidanzi B.E. (2018). Validation of Transition Readiness Assessment Questionaire in Turkish Adolescents with Diabetes. Balk. Med. J..

[46] Anelli C.G., Len C.A., Terreri M.T.R.A., Russo G.C.S., Reiff A.O. (2019). Translation and validation of the Transition Readiness Assessment Questionnaire (TRAQ). J. Pediatr..

[47] Philpott J.R., Kurowski J.A. (2019). Challenges in Transitional Care in Inflammatory Bowel Disease: A Review of the Current Literature in Transition Readiness and Outcomes. Inflamm. Bowel Dis..

[48] Johnson L.E., Lee M.J., Turner-Moore T., Tate L.R.G., Brooks A.J., Tattersall R.S., Jones G.L., Lobo A.J. (2021). Systematic Review of Factors Affecting Transition Readiness Skills in Patients with Inflammatory Bowel Disease. J. Crohns. Colitis..

[49] Ayuk A.C., Onukwuli V.O., Obumneme-Anyim I.J., Eze J.N., Akubuilo U.C., Mbanefo N.R., Iloh K.K., Ezenwosu O.U., Odetunde I.O., Okafor H.U. (2020). Pre-Transition Readiness in Adolescents and Young Adults with Four Chronic Medical Conditions in South East Nigeria An African Perspective to Adolescent Transition. Adolesc. Health Med..

[50] Eluri S., Book W.M., Kodroff E., Strobel M.J., Gebhart J.H., Jones P.D., Menard-Katcher P., Ferris M.E., Dellon E.S. (2017). Lack of Knowledge and Low Readiness for Health Care Transition in Eosinophilic Esophagitis and Eosinophilic Gastroenteritis. J. Pediatr. Gastroenterol. Nutr..

[51] Available Versions of the STARx Questionnaire. https://www.med.unc.edu/transition/transition-tools/trxansition-scale/versions-of-the-starx-questionnaire/.

[52] Casellas F., Ginard D., Vera I., Torrejón A., GETECCU (2013). Development and testing of a new instrument to measure patient satisfaction with health care in inflammatory bowel disease: The CACHE questionnaire. Inflamm. Bowel Dis..

