# Supplementary material for: Hungarian Linguistic, Cross-Cultural and Age Adaptation of Transition Specific Questionnaires in Patients with Inflammatory Bowel Disease
Source: Children (Basel). 2023 Apr 12;10(4):711. doi: 10.3390/children10040711 (PMC10137128; doi:10.3390/children10040711)
Supplement: Supplementary file 1 [file children-10-00711-s001.zip › Supplementary figure_S1.pdf]

Supplementary Figure S1 Steps of adaptation

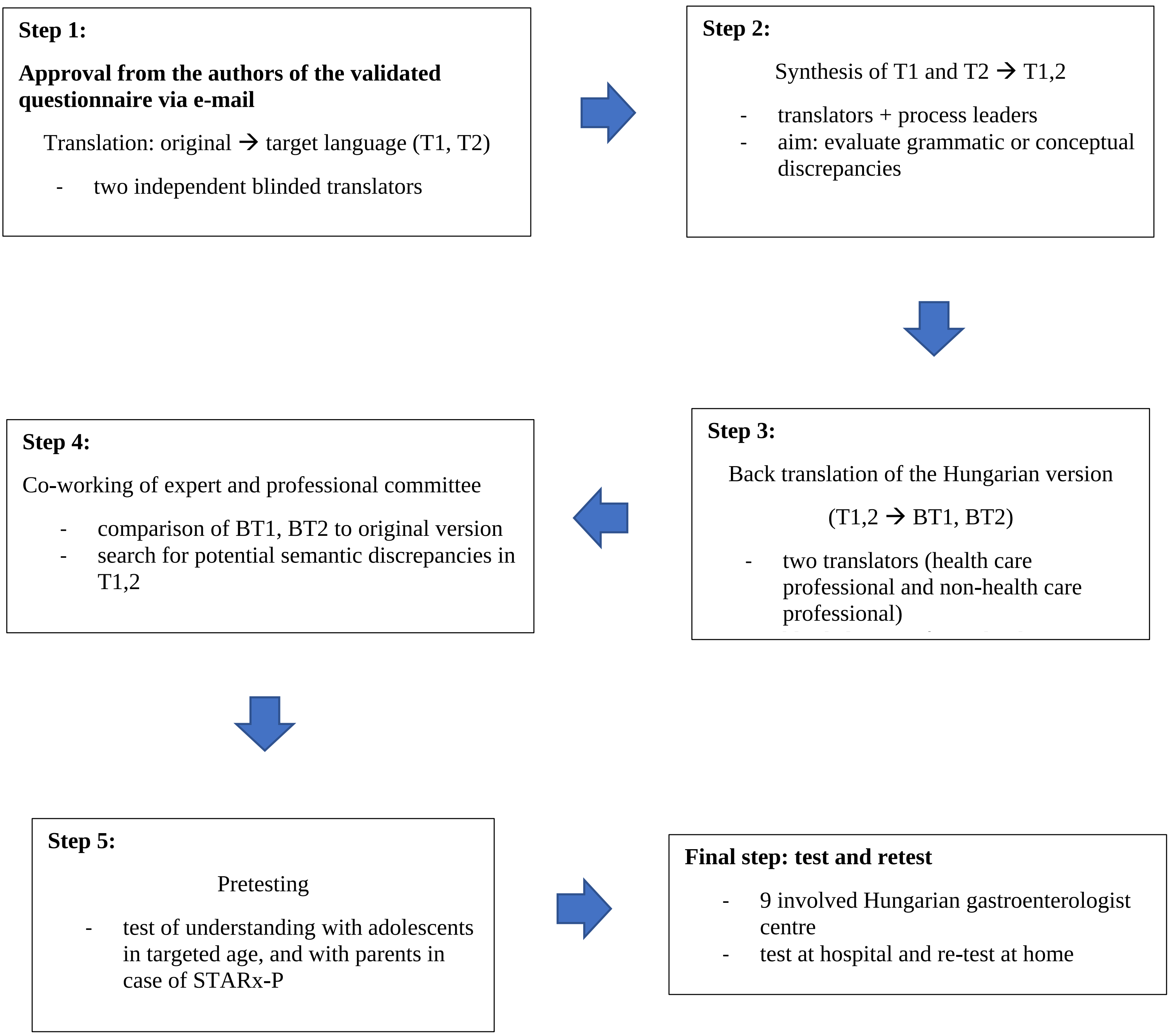

First, the surveys were prepared by two independent translators in the target Hungarian language (T1, T2). DD, PS and AE then compared the translated versions to evaluate any grammatic or conceptual discrepancies, and integrated them (T1,2). In the next step, the T1,2 was translated backward by two independent translators (BT1, BT2). The backtranslated versions were compared by DD, PS, AE, and language professionals to the original survey, searching for semantic discrepancies due to bidirectional translations. Afterwards, pre-test was performed with 30 adolescents by DD and AE in children's hospitals. In the last step, nine hospitals in Hungary were involved in completing tests and re-tests of adolescents. At least four to five individuals per question were included to fulfil the survey, and re-test was performed within 7-10 days. The second questionnaire was issued to participants in a stamped envelope.
