# Supplementary material for: Hungarian Linguistic, Cross-Cultural and Age Adaptation of Transition Specific Questionnaires in Patients with Inflammatory Bowel Disease
Source: Children (Basel). 2023 Apr 12;10(4):711. doi: 10.3390/children10040711 (PMC10137128; doi:10.3390/children10040711)
Supplement: Supplementary file 1 [file children-10-00711-s001.zip › Supplementary_table_S1.pdf]

Supplementary Table S1 The Hungarian version of the IBD-SES questionnaire

IBD Self-Efficacy Scale for adolescents and young adults
Gyulladásos bélbetegség énhatékonyság kérdőív (IBD-SES)

You can find **13 statements** on adolescent inflammatory bowel disease below. The statements are about how you deal with inflammatory bowel disease and your daily activities. For each statement, please select the answer that is the truest **for the past 2 weeks.**

Alább látható **13 állítás** a serdülőkori gyulladásos bélbetegségre vonatkozóan. Az állítások azzal kapcsolatosak, hogy hogyan kezeled a gyulladásos bélbetegséggel és a mindennapi életviteleddel kapcsolatos dolgokat.
Kérjük, hogy mindegyik állítás esetén válaszd ki azt a választ, amelyik leginkább igaz **az elmúlt 2 hétre**.

Original version	Hungarian version
**Do you agree with the following statements?**	**Egyetértesz a következő állításokkal?**
1. I understand what inflammatory bowel disease is	1. Értem, hogy mi a gyulladásos bélbetegég
2. If someone asked me, I could explain what a colonoscopy is for.	2. Ha valaki megkérdezné, el tudnám magyarázni neki, hogy mi az a vastagbéltükrözés (kolonoszkópia).
3. Remembering to take my IBD medications is hard.	3. Nehéz emlékezni rá, hogy bevegyem a gyulladásos bélbetegség miatt szedett gyógyszereimet.
4. I can get through my day, even if I have symptoms like abdominal pain or fatigue.	4. Át tudom vészelni a napot, még akkor is, ha olyan tüneteim vannak, mint a hasi fájdalom vagy a fáradtság.
5. I worry about how IBD will affect my future.	
6. When asked, I can remember the names of my current IBD medications and what they are used for	6. Ha megkérdik, emlékszem a gyulladásos bélbetegség miatt jelenleg szedett gyógyszereim nevére és hogy mire kapom őket.
7. When I am feeling frustrated about having IBD, I have someone I can turn to	7. Amikor csalódott vagyok a betegségem miatt, van kihez forduljak.
8. I feel comfortable talking to my IBD doctor about my questions or concerns.	8. Könnyedén beszélgetek a kezelőorvosommal a gyulladásos bélbetegséggel kapcsolatos kérdéseimről vagy aggályaimról.
9. No matter where I am, I can find foods that I can eat.	9. Mindegy, hol tartózkodom, találok olyan ételt, amit megehetek.
10. I know what to do when I think a flare is starting.	10. Tudom, mi a teendő, ha azt gondolom, hogy újra fellángolt a betegségem.
11. I know where to find a reliable answer if I don't understand what my IBD doctor tells me	11. Tudom, hol találhatok megbízható választ, ha nem értem, hogy mit mond a kezelőorvosom.
12. I know what will make me feel better even when I am sad, frustrated, scared, angry, or annoyed.	12. Tudom, hogy mitől fogom magam jobban érezni, ha szomorú, csalódott, rémült, dühös vagy bosszús vagyok
13. I am hopeful that my IBD symptoms will get better	

Casellas F et al. Development and testing of a new instrument to measure patient satisfaction with health care in inflammatory bowel disease: the CACHE questionnaire. Inflamm Bowel Dis. 2013 Mar;19(3):559-68.
