# Supplementary material for: Hungarian Linguistic, Cross-Cultural and Age Adaptation of Transition Specific Questionnaires in Patients with Inflammatory Bowel Disease
Source: Children (Basel). 2023 Apr 12;10(4):711. doi: 10.3390/children10040711 (PMC10137128; doi:10.3390/children10040711)
Supplement: Supplementary file 1 [file children-10-00711-s001.zip › Supplementary_table_S2.pdf]

Supplementary Table S2 The mean and the Cronbach's α values of the domains in IBD-SES

Domains	***mean values of patients' self-efficacy (SD)***	***Cronbach's α***
managing medical care	4.002 (±1.053)	0.585
managing everyday life with IBD	3.892 (±0.978)	0.472
managing feelings	4.004 (±1.039)	0.617

IBD: inflammatory bowel disease
