# Supplementary material for: Hungarian Linguistic, Cross-Cultural and Age Adaptation of Transition Specific Questionnaires in Patients with Inflammatory Bowel Disease
Source: Children (Basel). 2023 Apr 12;10(4):711. doi: 10.3390/children10040711 (PMC10137128; doi:10.3390/children10040711)
Supplement: Supplementary file 1 [file children-10-00711-s001.zip › Supplementary_table_S3.pdf]

Supplementary Table S3 Correlation between total and domain subscores of the questionnaires and demographic data

	p values of the questionnaires total score and domains	*gender*	*ethnicity*	*age at adaptation*[1]	*disease duration time*[1]	*disease type*	*previous intestinal surgery*	*number of medications*	*biological treatment*	*steroid treatment*	*AZA treatment*	*5-ASA treatment*	*comorbidities*
**IBD-SES**	*total score*	0.549	0.745	0.091	0.194	**0.016**	0.375	0.246	0.217	0.755	**0.008**	0.515	0.596
	'managing medical care'	0.364	0.869	0.075	0.412	**0.008**	0.082	0.955	0.372	0.617	0.069	0.925	0.451
	managing everyday life with IBD'	0.625	0.609	0.197	0.273	0.065	0.452	**0.034**	0.248	0.708	**0.003**	0.412	0.442
	managing feelings'	0.372	0.648	0.130	0.137	0.243	0.846	0.093	0.115	0.535	0.113	0.992	0.985
TRAQ	*total score*	0.401	0.536	**0.000**	0.994	0.871	0.754	0.672	0.413	0.352	0.882	0.302	0.832
	'managing medications'	0.830	0.367	**0.000**	0.227	0.927	0.705	0.140	0.630	0.545	0.704	0.055	0.710
	'appointment keeping'	0.576	0.148	**0.001**	0.933	0.515	0.635	0.905	0.772	0.313	0.797	0.488	0.899
	'tracking health issues'	**0.044**	0.731	**0.000**	0.627	0.664	0.643	0.260	**0.040**	0.221	0.100	0.913	0.836
	'talking with providers'	0.948	0.299	0.105	0.864	0.328	0.731	0.809	0.581	0.919	0.328	0.573	0.753
	'managing daily activities'	**0.004**	0.694	0.068	0.510	0.688	0.661	0.587	0.198	0.662	0.362	0.465	0.381
STARx-A	*total score*	0.506	0.763	0.194	0.445	0.387	0.586	0.600	0.971	0.559	0.122	0.504	0.526
	'medication management'	0.831	**0.034**	0.551	0.052	0.200	0.155	0.067	0.168	0.617	0.732	0.378	0.473
	'provider communication'	0.792	0.990	0.580	0.583	0.119	0.814	0.252	0.876	0.372	0.304	0.204	0.253
	'engagement during appointments'	0.780	0.935	0.350	**0.032**	0.050	0.801	0.380	0.995	0.185	0.221	0.346	0.726
	disease knowledge	0.119	0.213	0.988	0.246	0.333	0.319	0.795	0.759	0.578	0.630	0.679	0.685
	'adult health responsibilities'	0.758	0.942	0.201	0.661	0.175	0.196	0.923	0.218	0.276	0.982	0.592	0.556
	'resource utilization'	0.408	0.773	0.407	0.708	0.169	0.625	0.569	0.142	0.430	0.343	0.317	0.982

[1]: results are given in correlation coefficients (ρ)

Significant results are shown in bold

AZA: azathioprine; 5-ASA: 5-aminosalicylic acid
