# Supplementary material for: Hungarian Linguistic, Cross-Cultural and Age Adaptation of Transition Specific Questionnaires in Patients with Inflammatory Bowel Disease
Source: Children (Basel). 2023 Apr 12;10(4):711. doi: 10.3390/children10040711 (PMC10137128; doi:10.3390/children10040711)
Supplement: Supplementary file 1 [file children-10-00711-s001.zip › Supplementary_table_S4.pdf]

Supplementary Table S4 The Hungarian version of the TRAQ questionnaire

Transition Readiness Assessment Questionnaire (TRAQ)
Kérdőív a tranzíciós felkészültség megítélésére (TRAQ)

Please select one box in each row which is most characteristic of you with respect to your transition readiness in the different topics! There is no right or wrong answer

Alább látható **13 állítás** a serdülőkori gyulladásos bélbetegségre vonatkozóan. Az állítások azzal kapcsolatosak, hogy hogyan kezeled a gyulladásos bélbetegséggel és a mindennapi életviteleddel kapcsolatos dolgokat.
Kérjük, hogy mindegyik állítás esetén válaszd ki azt a választ, amelyik leginkább igaz **az elmúlt 2 hétre**.

Original version	Hungarian version
**Managing medications**	**Gyógyszerszedés**
1. Do you fill a prescription if you need to?	1. Feliratsz magadnak gyógyszert, ha szükséged van rá?
2. Do you know what to do if you are having a bad reaction to your medications?	2. Tudod-e mit kell tenned, ha egy gyógyszerre rosszul reagálsz? (pl. mellékhatás)
3. Do you take medications correctly and on your own?	3. Megfelelően és önállóan szeded a gyógyszereid?
4. Do you reorder medications before they run out?	4. Feliratod magadnak a gyógyszereid, mielőtt elfogynának?
**Appointment keeping**	**Időpontok menedzselése**
5. Do you call the doctor's office to make an appointment?	5. Te hívod fel az orvosi rendelőt (recepció), hogy időpontot egyeztess?
6. Do you follow up on any referral for tests or check-ups or labs?	6. Nyomon követed a vizsgálatok, ellenőrzések vagy laborvizsgálatok eredményét?
7. Do you arrange for your ride to medical appointments?	7. Magad szervezed meg az utazást az orvosi időpontokra?
8. Do you call the doctor about unusual changes in your health (e.g., allergic reactions)?	8. Értesíted a kezelőorvosod, ha szokatlan változást észlelsz az egészségi állapotodban? (pl. allergiás reakció)
9. Do you apply for health insurance if you lose your current coverage?	9. Ha lejár a tartós betegség igazolásod, kérelmezed-e a hosszabbítást?
10. Do you know what your health insurance covers?	10. Tudod, hogy milyen ellátást fedez az egészségbiztosításod?
11. Do you manage your money and budget household expenses (e.g., use checking/debit card)?	11. Önállóan gazdálkodsz a pénzeddel és fedezed a háztartási költségeket? (pl. van bankkártyád?)
**Tracking health issues**	**Az egészségügyi teendők dokumentálása**
12. Do you fill out the medical history form, including a list of your allergies?	12. Ki tudod tölteni a korábbi betegségeidre, illetve az allergiádra vonatkozó nyomtatványt?

Casellas F et al. Development and testing of a new instrument to measure patient satisfaction with health care in inflammatory bowel disease: the CACHE questionnaire. Inflamm Bowel Dis. 2013 Mar;19(3):559-68.

13. Do you keep a calendar or list of medical and other appointments?	13. Vezetsz nyilvántartást (pl. naptár) vagy feljegyzést az orvosi és egyéb vizsgálati időpontjaidról?
14. Do you make a list of questions before the doctor's visit?	14. Készítesz listát a kérdéseidről mielőtt orvoshoz mész?
15. Do you get financial help with school or work?	15. Kapsz pénzügyi támogatást az iskolában vagy a munkában? (pl. segély, ösztöndíj)
**Talking with providers**	**Kommunikáció az ellátó személyzettel**
16. Do you tell the doctor or nurse what you are feeling?	16. Megosztod az érzéseidet az orvosoddal és a nővérekkel?
17. Do you answer questions that are asked by the doctor, nurse or clinic staff?	17. Te válaszolod meg az orvos, a nővér vagy az egyéb egészségügyi személyzet (pl.: dietetikus) által feltett kérdéseket?
**Managing daily activities**	**Napi tevékenységek**
18. Do you help plan or prepare meals/food?	18. Segítesz kitalálni vagy elkészíteni az ételeket?
19. Do you keep home/room clean or clean up after meals?	19. Figyelsz otthon a tisztaságra? Étkezések után eltakarítasz magad után?
20. Do you use neighbourhood stores and services (e.g., grocery stores and pharmacy stores)?	20. Igénybe veszed a szomszédos kisüzleteket szolgáltatásait? (pl. élelmiszerboltok és gyógyszertárak)

Casellas F et al. Development and testing of a new instrument to measure patient satisfaction with health care in inflammatory bowel disease: the CACHE questionnaire. Inflamm Bowel Dis. 2013 Mar;19(3):559-68.
