# Supplementary material for: Hungarian Linguistic, Cross-Cultural and Age Adaptation of Transition Specific Questionnaires in Patients with Inflammatory Bowel Disease
Source: Children (Basel). 2023 Apr 12;10(4):711. doi: 10.3390/children10040711 (PMC10137128; doi:10.3390/children10040711)
Supplement: Supplementary file 1 [file children-10-00711-s001.zip › Supplementary_table_S5.pdf]

Supplementary Table S5 The mean and the Cronbach's α values of the domains in TRAQ

Domains	*mean values of patients' transition readiness (SD)*	*Cronbach's α*
managing medication	3.820 (±0.862)	0.724
appointment keeping	3.448 (±0.886)	0.827
tracking health issues	2.777 (±0.988)	0.546
talking with providers	3.005 (±1.105)	0.577
managing daily activities	3.751 (±0.930)	0.622
