# Supplementary material for: Hungarian Linguistic, Cross-Cultural and Age Adaptation of Transition Specific Questionnaires in Patients with Inflammatory Bowel Disease
Source: Children (Basel). 2023 Apr 12;10(4):711. doi: 10.3390/children10040711 (PMC10137128; doi:10.3390/children10040711)
Supplement: Supplementary file 1 [file children-10-00711-s001.zip › Supplementary_table_S6.pdf]

Supplementary Table S6 A The Hungarian version of the STARx-A questionnaire

STARx Transition Readiness Questionnaire (Adolescent Version)
STARx tranzíciós felkészültség kérdőív (serdülő példánya)

Original version	Hungarian version
**Section 1. Please check the one that shows how often you have done each thing in the past 3 months.**	**1. rész** Milyen gyakran végezted a következő dolgokat az elmúlt **3 hónapban?** Kérjük, jelöljd be mindegyik sorban azt az egy négyzetet, amelyik a legjellemzőbb!
1. How often did you make an effort to understand what your doctor told you?	1. Milyen gyakran törekedtél arra, hogy megértsd a kezelőorvos által mondottakat?
2. How often did you take your medicines on your own?	2. Milyen gyakran vetted be önállóan a gyógyszereid?
3. How often did you ask doctors or nurses questions about your illness, medicines or medical care?	3. Milyen gyakran tettél fel kérdéseket az kezelőorvosodnak vagy a nővéreknek a betegségeddel, gyógyszereiddel vagy az ellátásoddal kapcsolatban?
4. How often did you make your own appointments?	
5. How often did you need someone to remind you to take your medicines?	5. Milyen gyakran kellett emlékeztetni a gyógyszereid beszedésére?
6. How often did you use things like pillboxes, schedules, or alarm to help you take their medicines when they were supposed to?	6. Milyen gyakran használtál emlékeztetőt vagy ébresztőórát, hogy időben vedd be a gyógyszereidet?
7. How often did you use the internet, books or other guides to find out more about his/her illness?	7. Milyen gyakran használtál internetet, könyveket vagy egyéb útmutatókat, hogy tájékozódj a betegségeddel kapcsolatban?
8. How often did your forget to take your medicines?	8. Milyen gyakran felejtetted el bevenni a gyógyszereid?
9. How often did you work with your doctor to take care of new health problems that came up?	9. Milyen gyakran beszéltél az orvosoddal az újonnan fellépő egészségi problémák megoldásáról?
**Section 2. Some patients know a lot about their health and some patients do not. How much does you know? Please check the best answer.**	**2. rész** Egyes betegek sokat tudnak az egészségükről, egyesek viszont keveset. Te mennyit tudsz? Jelöld meg azt a válaszlehetőséget, amelyik a **mai napon** legjobban tükrözi a betegségeddel kapcsolatos tudásod!
10. How much do you know about your illness?	10. Mennyi mindent tudsz a betegségedről?
11. How much do you know about taking care of your illness?	11. Mit tudsz elmondani a betegséged kezeléséről?
12. How much do you know about what will happen if you do not take your medicines?	12. Mit tudsz arról, hogy mi fog történni, ha nem szeded rendesen a gyógyszereid?
**Section 3. Some patients may find it hard to do certain things. Please check what best describes how easy or hard you feel it will be for you.**	**3. rész** Néhány beteg nehezen tud bizonyos dolgokat végrehajtani. Neked mennyire könnyű vagy nehéz a következő dolgokat megcsinálnod? Jelöld meg azt a válaszlehetőséget, amelyik a **mai napon** legjobban tükrözi, hogy mennyire találod nehéznek az alábbiakat!
13. How easy or hard is it for you to talk to your doctor?	13. Menyire könnyű vagy nehéz az orvosoddal beszélgetni?
14. How easy or hard is it for you to make a plan with your doctor to care for your health?	14. Mennyire könnyű vagy nehéz az orvosoddal terveket készíteni az egészséged gondozásával kapcsolatban?
15. How easy or hard is it for you to see your doctor by yourself?	

Casellas F et al. Development and testing of a new instrument to measure patient satisfaction with health care in inflammatory bowel disease: the CACHE questionnaire. Inflamm Bowel Dis. 2013 Mar;19(3):559-68.

16. How easy or hard is it for you to take your medicines the way they are supposed to?	16. Mennyire könnyű vagy nehéz úgy szedni a gyógyszereidet, ahogy azokat az orvos előírja?
17. How easy or hard is it for you to take care of yourself?	17. Mennyire könnyű vagy nehéz magadról gondoskodnod?
18. How easy or hard do you think it will be for you to move from pediatrics to adult-focused care?	18. Mennyire lesz könnyű vagy nehéz a gyermekorvostól a felnőtt gasztroenterológushoz átkerülni?

Casellas F et al. Development and testing of a new instrument to measure patient satisfaction with health care in inflammatory bowel disease: the CACHE questionnaire. Inflamm Bowel Dis. 2013 Mar;19(3):559-68.

Supplementary table 6 B The Hungarian version of the STARx-P questionnaire

STARx Transition Readiness Questionnaire (ParentVersion)
STARx tranzíciós felkészültség kérdőív (szülő példánya)

Original version	Hungarian version
**Section 1. Please check the one that shows how often your child has done each thing in the past 3 months.**	**1. rész** Milyen gyakran végezted a következő dolgokat az elmúlt **3 hónapban?** Kérjük, jelöljd be mindegyik sorban azt az egy négyzetet, amelyik a legjellemzőbb!
1. How often did your child make an effort to understand what his/her doctor told them?	1. Milyen gyakran törekedett gyermeke arra, hogy megértse a kezelőorvos által mondottakat?
2. How often did your child take his/her medicines on their own?	2. Milyen gyakran vette be gyermeke önállóan a gyógyszereit?
3. How often did your child ask his/her doctor or nurse questions about their illness, medicines or medical care?	3. Milyen gyakran tett fel gyermeke kérdéseket az kezelőorvosnak vagy a nővéreknek a betegségével, gyógyszereivel vagy az ellátással kapcsolatban?
4. How often did your child make their own appointments?	
5. How often did your child need someone to remind him/her to take their medicines?	5. Milyen gyakran kellett valakinek emlékeztetni gyermekét a gyógyszerei beszedésére?
6. How often did your child use things likes pillboxes, schedules, or alarm clocks to help him/her take their medicines when they were supposed to?	6. Milyen gyakran használ gyermeke gyógyszerdobozt, emlékeztetőt vagy ébresztőórát, hogy időben bevegye a gyógyszereit?
7. How often did your child use the internet, books or other guides to find out more about his/her illness?	7. Milyen gyakran használ gyermeke internetet, könyveket vagy egyéb útmutatókat, hogy tájékozódjon a betegségével kapcsolatban?
8. How often did your child forget to take his/her medicines?	8. Milyen gyakran felejtette el gyermeke bevenni a gyógyszereit?
9. How often did your child work with his/her doctor to take care of new health problems that came up?	9. Milyen gyakran beszéli át gyermeke az újonnan fellépő egészségügyi problémáit az orvosával?
**Section 2. Some patients know a lot about their health and some patients do not. How much does your child know? Please check the answer that describes your child most.**	**2. rész** Egyes betegek sokat tudnak az egészségükről, egyesek viszont keveset. Te mennyit tudsz? Jelöld meg azt a válaszlehetőséget, amelyik a **mai napon** legjobban tükrözi a betegségeddel kapcsolatos tudásod!
10. How much does your child know about his/her illness?	10. Mennyi mindent tud gyermeke a betegségéről?
11. How much does your child know about taking care of his/her illness?	11. Mit tud elmondani gyermeke a betegsége kezeléséről?
12. How much does your child know about what will happen if he/she does not take their medicines?	12. Mit tud gyermeke arról, hogy mi fog történni, ha nem szedi a gyógyszereit?
**Section 3. Some patients may find it hard to do certain things. Please check what best describes how easy or hard you feel this is/will be for your child.**	**3. rész** Néhány beteg nehezen tud bizonyos dolgokat végrehajtani. Neked mennyire könnyű vagy nehéz a következő dolgokat megcsinálnod? Jelöld meg azt a válaszlehetőséget, amelyik a **mai napon** legjobban tükrözi, hogy mennyire találod nehéznek az alábbiakat!
13. How easy or hard is it for your child to talk to his/her doctor?	13. Menyire könnyű vagy nehéz gyermekének az orvosával beszélni?

Casellas F et al. Development and testing of a new instrument to measure patient satisfaction with health care in inflammatory bowel disease: the CACHE questionnaire. Inflamm Bowel Dis. 2013 Mar;19(3):559-68.

14. How easy or hard is it for your child to make a plan with his/her doctor to care for his/her health?	14. Mennyire könnyű vagy nehéz gyermekének az orvosával terveket készíteni az egészséggondozással kapcsolatban?
15. How easy or hard is it for your child to see his/her doctor by himself or herself?	
16. How easy or hard is it for your child to take his/her medicines like they are supposed to?	16. Mennyire könnyű vagy nehéz gyermekének úgy szedni a gyógyszereit, ahogy azok elő vannak írva?
17. How easy or hard is it for your child to take care of himself/herself?	17. Mennyire könnyű vagy nehéz gyermekének magáról gondoskodnia?
18. How easy or hard do you think it will be for your child to move from pediatrics to adult-focused care?	18. Mennyire lesz könnyű vagy nehéz gyermekének a gyermekellátásból a felnőtt ellátásba átkerülnie?

Casellas F et al. Development and testing of a new instrument to measure patient satisfaction with health care in inflammatory bowel disease: the CACHE questionnaire. Inflamm Bowel Dis. 2013 Mar;19(3):559-68.
