# Supplementary material for: Hungarian Linguistic, Cross-Cultural and Age Adaptation of Transition Specific Questionnaires in Patients with Inflammatory Bowel Disease
Source: Children (Basel). 2023 Apr 12;10(4):711. doi: 10.3390/children10040711 (PMC10137128; doi:10.3390/children10040711)
Supplement: Supplementary file 1 [file children-10-00711-s001.zip › Supplementary_table_S7.pdf]

Supplementary Table S7 The mean and the Cronbach's α values of the domains in STARx

Domains	*mean values of patients' and parents transition readiness (SD)*		*Cronbach's α*	
	STARx-A	STARx-P	STARx-A	STARx-P
medication management	3.475 (±1.479)	3.391 (±1.461)	0.457	0.449
provider communication	4.335 (±0.775)	4.208 (±0.809)	0.779	0.828
engagement during appointments	3.747 (±1.202)	3.823 (±1.095)	0.636	0.474
disease knowledge	4.393 (±0.908)	4.660 (±0.658)	0.575	0.720
adult health responsibilities	3.469 (±1.136)	3.535 (±1.169)	0.633	0.543
resource utilization	2.943 (±1.412)	3.095 (±1.442)	0.014	0.297
